# Phosphatidylserine liposomes for *Mycobacterium abscessus* infections management in people with cystic fibrosis non-eligible for CFTR modulators

**DOI:** 10.3389/fimmu.2026.1681558

**Published:** 2026-01-28

**Authors:** Tommaso Olimpieri, Noemi Poerio, Fabio Saliu, Nicola I. Lorè, Fabiana Ciciriello, Greta Ponsecchi, Federico Alghisi, Daniela M. Cirillo, Marco M. D’Andrea, Maurizio Fraziano

**Affiliations:** 1 Department of Biology, University of Rome Tor Vergata, Rome, Italy; 2 Emerging Bacteria Pathogens Unit, Istituto di Ricovero e Cura a Carattere Scientifico (IRCCS) Ospedale San Raffaele, Milan, Italy; 3 Pneumology and Cystic Fibrosis Unit, Bambino Gesù Children’s Hospital, Istituto di Ricovero e Cura a Carattere Scientifico (IRCCS), Rome, Italy

**Keywords:** cystic fibrosis, host-directed therapy, innate immunity, liposomes, *Mycobacterium abscessus*

## Abstract

We previously demonstrated that phosphatidylserine liposomes (PS-L) reduce inflammation and enhance intracellular killing of *Mycobacterium abscessus* (Mab) in infected human macrophages, with functional or pharmacologically inhibited cystic fibrosis conductance regulator (CFTR). Here, we evaluated the *in vitro* therapeutic potential of PS-L in macrophages from people with cystic fibrosis (pwCF), either under therapeutic regimen or not with CFTR modulator therapy Elexacaftor/Tezacaftor/Ivacaftor (ETI). Results show that PS-L exerted an anti-inflammatory effect in Mab infected macrophages, reducing TNF-α and IL-1β production and inducing IL-10 release at early and late time points, respectively. In addition, PS-L significantly increased antimycobacterial activity in macrophages from pwCF either undergoing or not ETI regimen. Importantly, in ETI-ineligible pwCF, PS-L alone still was capable to enhance a significant antimycobacterial response. Finally, PS-L combined with amikacin further enhanced intracellular bacterial clearance compared to single treatments. Altogether, these findings support PS-L as a promising host-directed therapy against Mab infection, particularly for pwCF who cannot benefit from ETI.

## 1 Introduction

Cystic fibrosis (CF) is an autosomal recessive disorder caused by mutations in the cystic fibrosis transmembrane conductance regulator (CFTR) gene. CFTR dysfunction impairs chloride and bicarbonate transport across epithelial cells, resulting in highly viscous mucus that limits mucociliary clearance and favors bacterial persistence, leading to chronic recurrent infections in the lungs and pancreatic ducts (1). These stimuli, together with gastric acids, proteases, and oxygen radicals, continuously activate airway epithelial cells and immune responses, driving excessive pro-inflammatory cytokine production and sustaining a cycle of apoptosis, cell stress, and inflammation (2).

Within this context, *Mycobacterium abscessus* (Mab), an intrinsically drug-resistant pathogen, has emerged as a major threat in people with CF (pwCF). Mab accounts for 2.6–13% of all non-tuberculous mycobacterial (NTM) pulmonary infections (3), ranks second in NTM-related pulmonary disease, and is becoming increasingly prominent in CF centers worldwide (4). Mab success as an opportunist pathogen in pwCF is mainly due to its intrinsic and acquired drug resistance to a wide spectrum of antibiotic classes (5).

According to the 2020 ATS/ERS/ESCMID/IDSA guidelines, treatment of Mab pulmonary disease requires at least three antibiotics for no less than 12 months (6). Such prolonged regimens not only promote resistance but is also very likely to induce drug intolerance, further reducing the already limited therapeutic options. Hence, novel therapeutic strategies are urgently needed to both limit resistance development and reduce treatment burden.

The high incidence of Mab infections in pwCF is also linked to the impact of CFTR dysfunction on innate immunity. During phagocytosis, macrophages rely on CFTR to transport chloride ions into the maturing phagosome, which provides the main counterion conductance necessary for the generation of the H^+^ gradients required for proper acidification. In macrophages, defective CFTR impairs this process, favoring intracellular survival of engulfed bacteria (7) and significantly reducing intracellular pathogen-killing capacity (8). Thus, defective mucociliary clearance combines with impaired macrophage function to drive persistent infection and chronic lung inflammation, fueling the vicious cycle of infection, inflammation, and tissue damage that underlies lung function decline in pwCF (2).

Since 2019, the triple-combination therapy Elexacaftor/Tezacaftor/Ivacaftor (ETI) has shown remarkable efficacy in rescuing F508del-CFTR protein function, allowing to target those CF sub-populations carrying either two copies of F508del-CFTR or heterozygous for F508del-CFTR and a gating, residual, or minimal function mutation on the second allele (9). Considering the CFTR-related phagocytosis impairment in CF macrophages, ETI administration not only improves lung function and respiratory-related pwCF quality of life, but it is also linked to a decrease in infection frequency (10). However, many pwCF remain ineligible due to CFTR genotypes unresponsive to modulators, emphasizing the need for alternative therapeutic options.

In this context, multidrug-resistant (MDR) infections management remains a major unmet need, highlighting the urge for novel antibacterial agents and other immunotherapeutic options. We have developed a host-directed approach based on bioactive liposomes carrying lipid second messengers involved in phagocytosis, a crucial defense mechanism often subverted by Mab and other intracellular pathogens (11–13). We previously showed that phosphatidylserine liposomes (PS-L) enhance the bactericidal capacity of Mab-infected macrophages with pharmacologically inhibited CFTR, restore phagosome acidification and ROS production, and simultaneously dampen excessive inflammation via reduced NF-κB activation and TNF-α secretion (8). Moreover, liposomes carrying phosphatidylinositol 5-phosphate (ABL/PI5P) reduced intracellular Mab replication in CF macrophages, irrespective of ETI treatment (14).

Building on these findings, the present study investigates the *in vitro* immunotherapeutic potential of PS-L in Mab-infected CF macrophages, both in the presence and absence of ETI. We also assessed the added benefit of combining PS-L with amikacin, a host- and pathogen-directed strategy, that could provide new treatment opportunities for pwCF ineligible for ETI and contribute to reducing both the spread of further antimicrobial resistances and antibiotic treatment duration.

## 2 Materials and methods

### 2.1 Ethic statement

Cystic fibrosis patients, giving their (or parental) written informed consent to participate in the study, were enrolled at “Bambino Gesù” Children’s Hospital in Rome after having received detailed information on the scope and objectives of the study by sanitary personnel, who explained the patient information leaflet (ethics approval #738/2017 of “Bambino Gesù” Children’s Hospital, Rome).

### 2.2 pwCF

pwCF (*n* = 47) were enrolled at “Bambino Gesù” Children’s Hospital in Rome, Italy. All pwCF were clinically stable at the time of blood donation (5 ml). Clinical and demographic features of pwCF are summarized in **Supplementary Tables S1** and **S2**. Peripheral blood mononuclear cells were isolated by Ficoll density gradient, and monocytes were then positively sorted using anti-CD14 monoclonal antibodies conjugated to magnetic microbeads (Miltenyi Biotec), according to manufacturer’s instructions. Monocytes were then suspended 10^6^ cells/mL in RPMI 1640 supplemented with FBS 10% and L-glutammine 5 mM (all from Euroclone) and seeded in 96-well plates. Cells were differentiated in monocyte-derived macrophages (MDM) via stimulation with macrophage colony-stimulating factor (M-CSF) 50 ng/mL for 5 days.

### 2.3 Liposome preparation

PS-L liposomes were generated via thin layer evaporation technique. Briefly, 35 µg of L-α-phosphatidylserine (Avanti Polar Lipids) were dissolved in trichloromethane and 4 hrs organic solvent evaporation under vacuum at 42°C was carried out via Rotavapor^©^ R-100 (Büchi). Resulting lipid film was hydrated in 1 mL of ultrapure bi-distilled water (Millipore, Merck) by vortex mixing for 10 mins followed by 10 mins of sonication in a sonicating bath. Finally, to achieve a uniform liposome suspension in terms of vesicles dimension, liposomes were extruded 10 times through 0.22 µm polycarbonate membrane (mini-extruder, Avanti Polar Lipids).

### 2.4 Bacteria

*M. abscessus* reference strain American Type Culture Collection (ATCC) 19977 and the already described *M. abscessus* subsp *abscessus* clinical strain Mab285 were used (14). Mab and Mab285 single colonies were collected by streaking on Middlebrook 7H10 medium (7H10 - BD Difco™) supplemented with oleic acid, albumin, dextrose, and catalase (OADC), then suspended in 15 ml of Middlebrook 7H9 broth (7H9 - BD Difco™) supplemented with albumin, dextrose, catalase (ADC), and Tween 80 0.05%, and grown in Erlenmeyer flask at 37 °C under stirring for 40 hours. Growth was monitored by measuring the optical density at the wavelength of 600nm by a spectrophotometer (Varioskan LUX Multimode Microplate Reader, Thermo Fisher Scientific).

### 2.5 *in vitro* extracellular/intracellular mycobacterial growth evaluation

To assess the intracellular bacterial growth, MDM from pwCF were pre-stimulated with Elexacaftor 5 µM + Tezacaftor 5 µM + Ivacaftor 1 µM (ETI) for two days and then infected with Mab, for 3 hours at 37 °C at a multiplicity of infection (MOI) of 10. Thereafter, extracellular bacilli were killed by 1 hour incubation with amikacin 250 µg/ml. Cells were then washed and incubated with PS-L (525ng/ml) and/or ETI for 18 hours. Finally, cells were lysed with 1% deoxycholate (Sigma), diluted in PBS-Tween 80 0,05% and CFU quantified by plating bacilli in triplicate on 7H10. To evaluate the *in vitro* efficacy of a combined therapy on extracellular and intracellular mycobacterial viability, MDM from pwCF were infected with either Mab or Mab285 at MOI 10 for 3 hours at 37°C. Cells were then stimulated with PS-L and/or amikacin 4µg/ml (Amk) for 18 hours. Both extracellular and intracellular bacterial growth were assessed by plating on 7H10 agar. Replication indexes were calculated as the ratio between the CFU obtained after 18 hours from infection, in the presence or absence of stimuli, and those obtained immediately after the infection, before stimulation.

### 2.6 Enzyme-linked immunosorbent assay

For tumor necrosis factor-α (TNF-α), interleukin (IL) 1β, and IL-10 levels quantification, MDMs were infected or not with Mab as described in paragraph 2.5. After infection extracellular bacilli were removed by 1 hour incubation with amikacin 250 µg/ml and finally stimulated or not with PS-L (525ng/ml) for 3 or 18 hrs. Thereafter, supernatants were collected, all possible remaining bacteria or cellular debris removed by 5 mins centrifugation at 14000x*g*, and finally samples were stored at -20°C until analysis. The levels of TNF-α, IL-1β, and IL-10 were measured by human TNF-α, IL-1β, or IL-10 DuoSet^®^ ELISA Development Systems (R&D Systems, Minneapolis, MN, USA) as per manufacturer’s instructions.

### 2.7 Statistics

**Figure 1:**
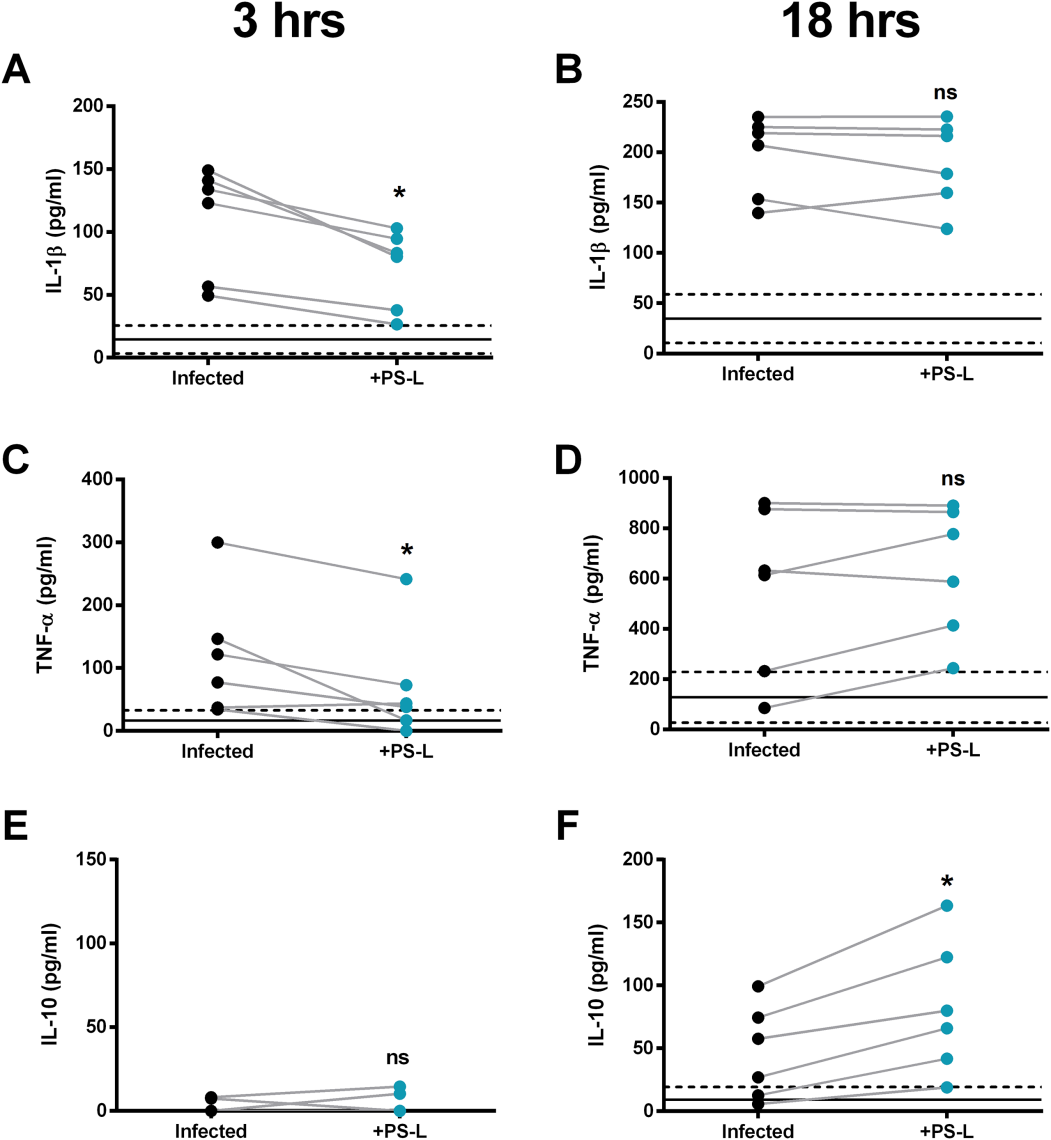
PS-L beneficially modulates pro- and anti-inflammatory cytokines IL-1β, TNF-α, and IL-10 production over the course of Mab infection on pwCF macrophages. MDM from pwCF (*n* = 6) were cultured at the concentration of 1x10^6^ cells/ml in 96-well plates. Cells were infected or not with Mab at MOI 10 for 3 hours at 37°C and then extracellular bacilli were killed by 1 hour incubation with amikacin 250µg/ml. Cells were finally stimulated or not with PS-L for 3 (A, C, E) or 18 hours (B, D, F) and supernatants harvested and stored at -20 °C until analysis. The production of IL-1β, TNF-α, and IL-10 was analyzed by ELISA as per manufacturer’s instructions. Baseline cytokines range levels from uninfected and untreated controls are displayed in the background as mean ± 95% CI. Statistical analysis was performed by using two-sided Wilcoxon matched-pairs signed rank test. ns = not significant; *p<0.05.

**Figure 2:**
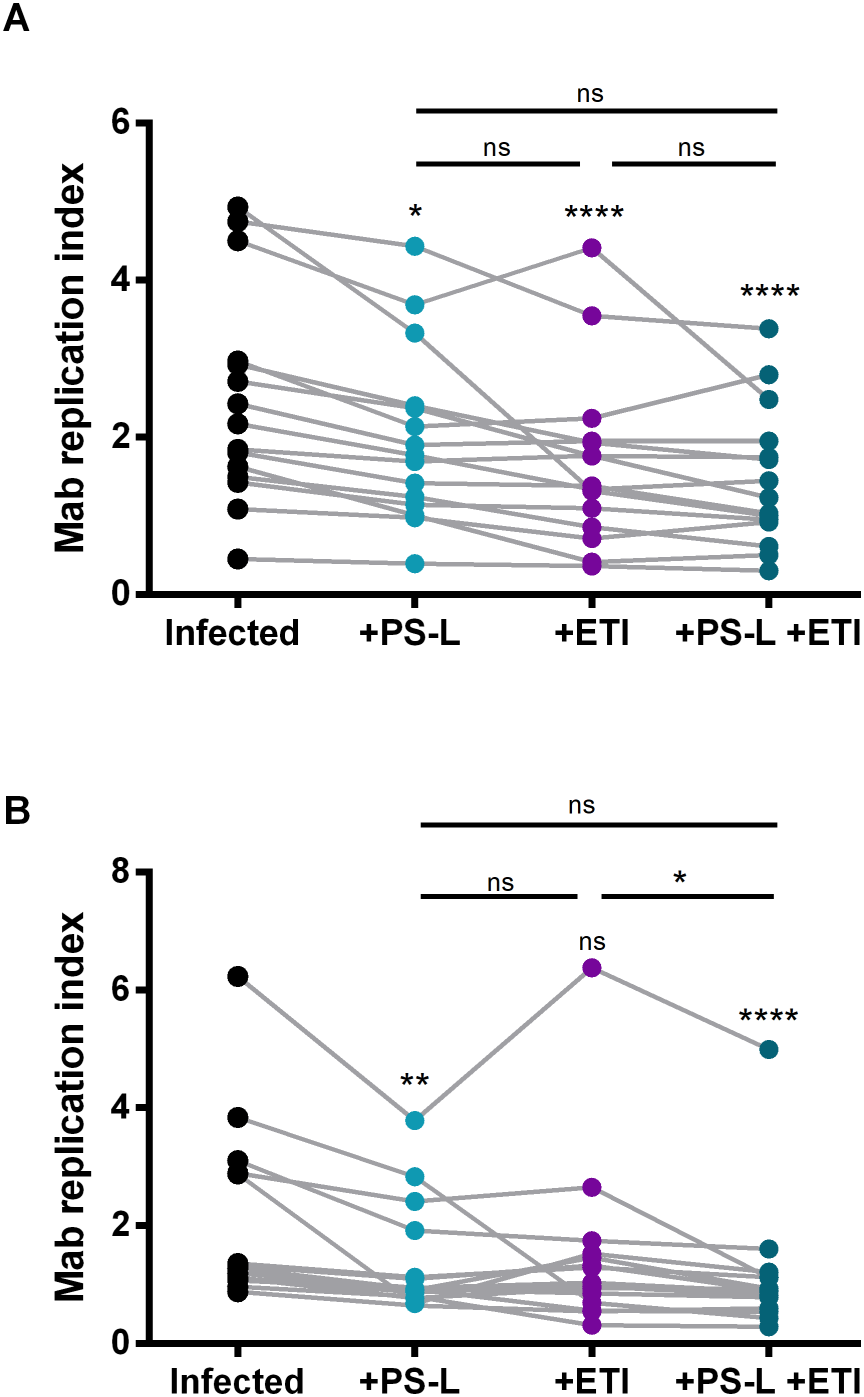
PS-L reduces Mab intracellular viability in macrophages from pwCF irrespectively for ETI eligibility. MDM from pwCF under ETI regimen (*n* = 15) **(A)** or from pwCF without any modulator regimen (*n* = 15) **(B)** were cultured at the concentration of 1x10^6^ cells/ml in 96-well plates. Cells were then infected with Mab at MOI 10 for 3 hours and finally stimulated with PS-L and/or ETI for 18 hours. Replication index was calculated as the ratio between the CFU obtained after 18 hours from infection, in the presence or absence of stimuli, and those obtained immediately after the infection. Statistical analysis was performed by using Fridman test followed by Dunn’s multiple comparisons test. ns = not significant; *p<0.05, **p<0.01, ****p<0.0001. If not indicated by the line, the comparisons were performed versus control.

**Figure 3:**
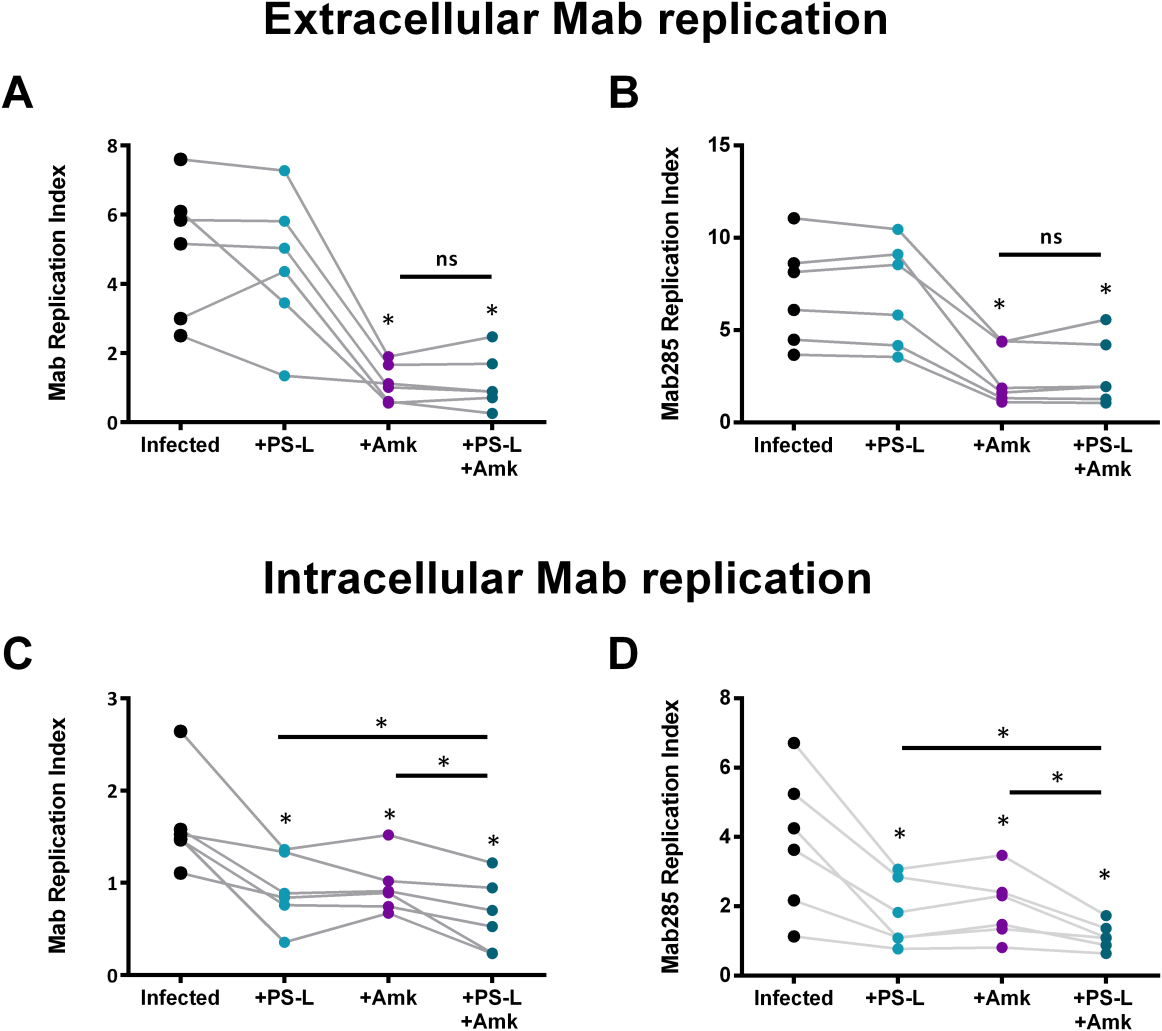
PS-L – amikacin combined treatment reduces both Mab and Mab285 intracellular viability in macrophages of ETI non-eligible pwCF. MDM from pwCF (*n* = 11) were cultured at the concentration of 1x10^6^ cells/ml in 96-well plates. Cells were infected with Mab **(A, C)** or Mab285 **(B, D)** at MOI 10 for 3 hours and stimulated with PS-L and/or amikacin 4µg/ml (Amk) for 18 hours. Finally, supernatants were collected, and MDM were lysed to enumerate extracellular **(A, B)** and intracellular **(C, D)** bacteria. Replication index was calculated as the ratio between the CFU obtained 18 hours after infection in the presence or absence of PS-L and/or Amk, and those obtained immediately after infection and before the stimuli. Statistical analysis was performed by using two-sided Wilcoxon matched-pairs signed rank test followed by Benjamini–Hochberg FDR correction. ns = not significant, *p<0.05. If not indicated by the line, the comparisons were performed versus control.

Statistical significance for comparisons between two groups was assessed using the two-sided Wilcoxon rank-sum test (**Figure 1** and **Supplementary Figure S1**). For analysis involving more than two related patient groups, statistical significance was determined by the Friedman test followed by Dunn’s *post-hoc* multiple comparisons on ranks with Bonferroni adjustment (**Figure 2**). For analysis involved a limited number of patients (n=6), pairwise comparisons between multiple patient groups were performed using the two-sided Wilcoxon rank-sum test, and the resulting p-values were adjusted using the Benjamini–Hochberg procedure to control the false discovery rate (FDR) (**Figure 3**).

## 3 Results

### 3.1 PS-L beneficially modulates pro- and anti-inflammatory cytokines IL-1β, TNF-α, and IL-10 production over the course of Mab infection on pwCF macrophages

Chronic and unresolved acute Mab infections in pwCF can cause progressive inflammatory lung damage (15, 16) especially, in those patients that cannot benefit from ETI treatment. Given the widely known anti-inflammatory function of PS (17), and considering the previous results regarding anti-inflammatory effects of liposomes containing PS (11, 12, 18), we investigated the effects of PS-L stimulation on pro- and anti-inflammatory cytokines IL-1β (**Figures 1A, B**), TNF-α (**Figures 1B, C**), and IL-10 (**Figures 1D, E**) production of CF macrophages from pwCF not receiving ETI therapeutic regimen (**Supplementary Table S1**) infected or not with Mab. Considering also that different cytokines have different time kinetics (19, 20), the quantification was carried out at two different time points, namely 3 and 18 hours post infection (**Figures 1A–F** respectively). For those cytokines with a short peak production time (IL-1β and TNF-α), PS-L stimulation caused a significant reduction in production levels at 3hrs post-infection which then reached plateau at 18hrs. On the other hand, IL-10, which displays a longer peak production time, could not be detected from any experimental condition in the short time frame, but its production resulted significantly enhanced by PS-L administration after 18hrs from infection when compared to infected and untreated controls.

### 3.2 PS-L reduces Mab intracellular viability in ETI-non receiving pwCF macrophages

To assess ETI impact on PS-L induced antimycobacterial response, MDM from pwCF either receiving (**Figure 2A**) or not (**Figure 2B**) ETI regimen (**Supplementary Tables S2A, B**) were *in vitro* infected with Mab and then treated with PS-L and/or ETI. **Figure 2A** shows that either ETI, PS-L, or their combination significantly enhance intracellular Mab clearance when compared to infected but untreated controls, even if no additive, synergic or interference effect could be detected when comparing PS-L/ETI combined treatment versus single PS-L or ETI administration.

Similarly, the *in vitro* efficacy of PS-L and/or ETI in MDM from pwCF currently not receiving ETI was evaluated (**Figure 2B**). This group comprises not only those pwCF who: i) possess the F508del mutation and do not receive ETI because of age, treatment refusal or waiting for the drug prescription, ii) have a mutation considered ETI-eligible only in the United States (21), iii) have a mutation which is currently under trial in Europe (EudraCT number:2021-005914-33); but also pwCF whose mutations are considered incompatible with ETI both in the US and EU.

**Figure 2B** shows that ETI is no longer able to reduce Mab intracellular viability, while PS-L retains its activity.

As supporting data regarding ETI-eligibility in pwCF not receiving treatment, **Supplementary Figure S1** shows the different responses to ETI (**Supplementary Figures S1A, C**) or PS-L (**Supplementary Figures S1B, D**) administration that ETI-eligible (**Supplementary Figures S1A, B**) vs ETI-non eligible (**Supplementary Figures S1C, D**) pwCF MDM display in terms of Mab intracellular replication reduction.

As expected, ETI *in vitro* treatment induces a significant reduction of Mab intracellular viability only in the ETI-eligible subgroup (**Supplementary Figure S1A**), while no effect was observed in the ETI-non eligible subgroup (**Supplementary Figure S1C**). Conversely and more importantly, PS-L treatment can reduce Mab intracellular viability in macrophages from pwCF not receiving (**Figure 2B**) ETI, and irrespectively of their eligibility status for the drugs (**Supplementary Figures S1B, D**).

### 3.3 PS-L – amikacin combined treatment reduces both Mab and Mab285 intracellular viability in macrophages of ETI non-eligible pwCF

The combined treatment PS-L - Amk promotes a higher reduction of both intracellular Mab and Mab285 viability in CF macrophages compared to single treatments. As a combined therapy based on antibiotic and bioactive liposomes may represent a valuable strategy to differentially target extracellular and intracellular pathogens (12, 14), we tested its efficacy in improving the mycobactericidal activity in Mab or Mab285-infected CF macrophages from pwCF who cannot benefit from ETI (**Supplementary Table S3**). Results in **Figure 3** show that PS-L has no direct effect on the extracellular Mab or Mab285 (**Figures 3A, B**), whereas significantly reduces the intracellular viability of both strains (**Figures 3C, D**). Furthermore, the combined treatment with PS-L and Amk (**Figures 3C, D**) induces a significant higher reduction of intracellular Mab or Mab285 replication index when compared to single treatments.

## 4 Discussion

Infectious diseases caused by MDR pathogens are a major global health concern (22). Most MDR infections occur in nosocomial settings and disproportionately affect immunocompromised individuals, including pwCF (23). These infections are particularly difficult to manage because of the limited number of effective antimicrobials (24), often leading to chronic and recurrent disease. In addition, MDR pathogens trigger sustained inflammatory responses that can damage host tissues, highlighting the importance of balancing pro- and anti-inflammatory cytokine production to limit tissue injury and promote repair (25, 26).

In recent years, host-directed therapies (HDTs) have emerged as a promising strategy to enhance host immunity and counteract pathogen-driven mechanisms of persistence. Among innate defense processes, phagocytosis represents a central mechanism that can be reinforced by HDT (24, 27). This process requires the timely regulation of lipid second messengers, which coordinate signal transduction, cytoskeleton remodeling, and membrane trafficking events (28, 29). For example, phosphatidylserine (PS) plays a key role in phagosome biology: its accumulation within the phagosome may alter its charge allowing the recruiting of proteins involved in membrane fusion and maturation, including synaptotagmins and small GTPases (28, 30). Not surprisingly, several intracellular pathogens, such as *Mycobacterium tuberculosis*, *Salmonella enterica*, *Legionella pneumophila*, and *Listeria monocytogenes*, exploit host lipid metabolism to block phagosome maturation and ensure survival (28, 31, 32). In this context, *Mycobacterium abscessus* (Mab) employs multiple strategies, including inhibition of phagosome acidification and membrane rupture mediated by ESX-4, to escape host killing (33).

Mab poses a particular challenge in pwCF, as it combines intrinsic resistance mechanisms, such as a waxy cell wall, efflux pumps, and drug-modifying enzymes, with the ability to rapidly acquire new resistances (34). Current Mab treatment regimens are long, complex, and frequently associated with toxicity, low patient adherence, and further resistance development (35, 36). The need for alternative therapeutic strategies is therefore urgent. In pwCF, CFTR dysfunction further compromises macrophage function. Loss of CFTR impairs PI3K/AKT signaling and blocks phagosomal acidification, thereby weakening bacterial clearance (7, 37, 38). This dual impairment (host-related phagocytosis defects and pathogen-driven phagosome escape) explains the severity of Mab infections in CF. Consequently, therapeutic strategies aimed at restoring phagosome maturation represent a rational and innovative approach.

We previously showed that PS-liposomes (PS-L) enhance mycobactericidal activity in Mab-infected macrophages by restoring phagosome acidification and ROS production, while simultaneously reducing NF-κB activation and TNF-α secretion (8). Here, we demonstrate that PS-L administration might be beneficial for the rebalancing of the inflammatory microenvironment during chronic Mab infections (**Figure 1**). A single treatment with our liposomes was able to significantly delay the production of IL-1β and TNF-α, while at the same time inducing IL-10 release. Such results, if projected to a treatment regimen instead of single dose, may result in a healthier lung milieu with reduced inflammation-caused tissue damage. Evidence supporting such speculations has been already partially acquired in a Mab chronically infected mice model, in which a treatment regimen consisting of injections of ABL/PI5P twice per week resulted in reduced levels of murine pro-inflammatory cytokines IL-1β and IFN-γ (12). PS-L treatment rebalances the inflammatory milieu by delaying IL-1β and TNF-α release while inducing IL-10 production. Although these findings derive from single-dose experiments, they suggest that repeated administration could foster a less damaging inflammatory environment, consistent with results obtained in Mab-infected mice treated with ABL/PI5P (12).

The introduction of the CFTR modulator Elexacaftor/Tezacaftor/Ivacaftor (ETI) has markedly improved outcomes for pwCF carrying at least one F508del allele (10). Nevertheless, a substantial proportion of patients remain ineligible due to non-responsive mutations, with eligibility rates significantly lower in Italy (≈70%) than in the US (≈90%) (39). Importantly, our data show that PS-L treatment promotes mycobactericidal activity in macrophages from pwCF independently of ETI treatment or eligibility (**Figures 2B** and **S1B, D**). Although PS-L combined with ETI did not significantly outperform single treatments (**Figures 2A, B**), these results identify PS-L as a valuable therapeutic option for ETI-ineligible patients.

Moreover, combining PS-L with amikacin (Amk) significantly enhanced the killing of both reference and clinical Mab strains in CF macrophages not receiving ETI (**Figure 3**). These findings are consistent with our previous demonstrations that ABL/PI5P synergizes with Amk in both *in vitro* and *in vivo* models (12, 14). Together, they support the concept of combined host- and pathogen-directed therapy to simultaneously target intracellular and extracellular Mab.

In conclusion our results validate, in an *ex vivo* experimental model, PS-L as a promising host-directed therapeutic tool for Mab infections. By enhancing phagosome maturation, restoring mycobactericidal activity, and rebalancing inflammatory responses, PS-L may offer an innovative adjunct or alternative to conventional antibiotics, particularly for pwCF who cannot benefit from ETI. Furthermore, its use in combination with antibiotics could improve infection control, reduce treatment duration, and help mitigate the spread of MDR pathogens.

## Data Availability

The original contributions presented in the study are included in the article/**Supplementary Material**. Further inquiries can be directed to the corresponding author.

## References

[1] Cutting GR . Cystic fibrosis genetics: from molecular understanding to clinical application. Nat Rev Genet. (2015) 16:45–56. doi: 10.1038/nrg3849, PMID: 25404111 PMC4364438

[2] Cohen-Cymberknoh M Kerem E Ferkol T Elizur A . Airway inflammation in cystic fibrosis: molecular mechanisms and clinical implications. Thorax. (2013) 68:1157–62. doi: 10.1136/thoraxjnl-2013-203204, PMID: 23704228

[3] Dedrick RM Abad L Storey N Kaganovsky AM Smith BE Aull HA . The problem of Mycobacterium abscessus complex: multi-drug resistance, bacteriophage susceptibility and potential healthcare transmission. Clin Microbiol Infect. (2023) 29:1335. doi: 10.1016/j.cmi.2023.06.026, PMID: 37364635 PMC10583746

[4] Degiacomi G Sammartino JC Chiarelli LR Riabova O Makarov V Pasca MR . Mycobacterium abscessus, an Emerging and Worrisome Pathogen among Cystic Fibrosis Patients. Int J Mol Sci. (2019) 20:5868. doi: 10.3390/ijms20235868, PMID: 31766758 PMC6928860

[5] Wu ML Aziz DB Dartois V Dick T . NTM drug discovery: status, gaps and the way forward. Drug Discov Today. (2018) 23:1502–19. doi: 10.1016/j.drudis.2018.04.001, PMID: 29635026 PMC6078814

[6] Daley CL Iaccarino JM Lange C Cambau E Wallace RJ Andrejak C . Treatment of nontuberculous mycobacterial pulmonary disease: an official ATS/ERS/ESCMID/IDSA clinical practice guideline. Eur Respir J. (2020) 56:2000535. doi: 10.1183/13993003.00535-2020, PMID: 32636299 PMC8375621

[7] Di A Brown ME Deriy LV Li C Szeto FL Chen Y . CFTR regulates phagosome acidification in macrophages and alters bactericidal activity. Nat Cell Biol. (2006) 8:933–44. doi: 10.1038/ncb1456, PMID: 16921366

[8] Olimpieri T Poerio N Ponsecchi G Di Lallo G D’Andrea MM Fraziano M . Phosphatidylserine liposomes induce a phagosome acidification-dependent and ROS-mediated intracellular killing of Mycobacterium abscessus in human macrophages. Front Cell Infect Microbiol. (2024) 14:1443719. doi: 10.3389/fcimb.2024.1443719, PMID: 39224705 PMC11366698

[9] Keating D Marigowda G Burr L Daines C Mall MA McKone EF . VX-445-tezacaftor-ivacaftor in patients with cystic fibrosis and one or two phe508del alleles. N Engl J Med. (2018) 379:1612–20. doi: 10.1056/NEJMoa1807120, PMID: 30334692 PMC6289290

[10] Connett G Maguire S Larcombe T Scanlan N Shinde S Muthukumarana T . Real-world impact of Elexacaftor-Tezacaftor-Ivacaftor treatment in young people with Cystic Fibrosis: A longitudinal study. Respir Med. (2025) 236:107882. doi: 10.1016/j.rmed.2024.107882, PMID: 39581272

[11] Poerio N De Santis F Rossi A Ranucci S De Fino I Henriquez A . Liposomes loaded with phosphatidylinositol 5-phosphate improve the antimicrobial response to pseudomonas aeruginosa in impaired macrophages from cystic fibrosis patients and limit airway inflammatory response. Front Immunol. (2020) 11:532225. doi: 10.3389/fimmu.2020.532225, PMID: 33117337 PMC7562816

[12] Poerio N Riva C Olimpieri T Rossi M Lorè NI De Santis F . Combined host- and pathogen-directed therapy for the control of mycobacterium abscessus infection. Microbiol Spectr. (2022) 10:e02546–21. doi: 10.1128/spectrum.02546-21, PMID: 35080463 PMC8791191

[13] Roux AL Viljoen A Bah A Simeone R Bernut A Laencina L . The distinct fate of smooth and rough Mycobacterium abscessus variants inside macrophages. Open biol. (2016) 6(11):160185. doi: 10.1098/rsob.160185, PMID: 27906132 PMC5133439

[14] Olimpieri T Poerio N Saliu F Lorè NI Ciciriello F Ponsecchi G . Phosphatidylinositol 5-phosphate-loaded apoptotic body-like liposomes for mycobacterium abscessus infection management in patients with cystic fibrosis. J Infect Dis. (2025): 232:e43-e47. doi: 10.1093/infdis/jiaf124, PMID: 40249250 PMC12308664

[15] Esther CR Esserman DA Gilligan P Kerr A Noone PG . Chronic Mycobacterium abscessus infection and lung function decline in cystic fibrosis. J Cyst Fibros: Off J Eur Cyst Fibros Soc. (2010) 9:117–23. doi: 10.1016/j.jcf.2009.12.001, PMID: 20071249 PMC3837580

[16] Floto RA Olivier KN Saiman L Daley CL Herrmann JL Nick JA . US Cystic Fibrosis Foundation and European Cystic Fibrosis Society consensus recommendations for the management of non-tuberculous mycobacteria in individuals with cystic fibrosis. Thorax. (2016) 71 Suppl 1:i1–22. doi: 10.1136/thoraxjnl-2015-207360, PMID: 26666259 PMC4717371

[17] Nagata S . Apoptosis and clearance of apoptotic cells. Annu Rev Immunol. (2018) 36:489–517. doi: 10.1146/annurev-immunol-042617-053010, PMID: 29400998

[18] Greco E Quintiliani G Santucci MB Serafino A Ciccaglione AR Marcantonio C . Janus-faced liposomes enhance antimicrobial innate immune response in Mycobacterium tuberculosis infection. Proc Natl Acad Sci United States America. (2012) 109:E1360–8. doi: 10.1073/pnas.1200484109, PMID: 22538807 PMC3361443

[19] Jönsson B Ridell M Wold AE . Phagocytosis and cytokine response to rough and smooth colony variants of Mycobacterium abscessus by human peripheral blood mononuclear cells. APMIS. (2013) 121:45–55. doi: 10.1111/j.1600-0463.2012.02932.x, PMID: 23030647

[20] Lee HM Yuk JM Kim KH Jang J Kang G Park JB . Mycobacterium abscessus activates the NLRP3 inflammasome via Dectin-1-Syk and p62/SQSTM1. Immunol Cell Biol. (2012) 90:601–10. doi: 10.1038/icb.2011.72, PMID: 21876553 PMC3389799

[21] Vertex Pharmaceuticals . Trikafta, Highlights of Prescribing Information (2024). Available online at: https://pi.vrtx.com/files/uspi_elexacaftor_tezacaftor_ivacaftor.pdf (Accessed November 8, 2024).

[22] Bassetti M Righi E Carnelutti A Graziano E Russo A . Multidrug-resistant klebsiella pneumoniae: Challenges for treatment, prevention and infection control. Expert Rev Anti-Infective Ther. (2018) 16:749–61. doi: 10.1080/14787210.2018.1522249, PMID: 30207815

[23] Duhaniuc A Păduraru D Nastase EV Trofin F Iancu LS Sima CM . Multidrug-resistant bacteria in immunocompromised patients. Pharm (Basel). (2024) 17:1151. doi: 10.3390/ph17091151, PMID: 39338313 PMC11434862

[24] Zumla A Rao M Wallis RS Kaufmann SHE Rustomjee R Mwaba P . Host-directed therapies for infectious diseases: Current status, recent progress, and future prospects. Lancet Infect Dis. (2016) 16:e47–63. doi: 10.1016/S1473-3099(16)00078-5, PMID: 27036359 PMC7164794

[25] Kany S Vollrath JT Relja B . Cytokines in inflammatory disease. Int J Mol Sci. (2019) 20:6008. doi: 10.3390/ijms20236008, PMID: 31795299 PMC6929211

[26] Opal SM DePalo VA . Anti-inflammatory cytokines. Chest. (2000) 117:1162–72. doi: 10.1378/chest.117.4.1162, PMID: 10767254

[27] Kaufmann SHE Dorhoi A Hotchkiss RS Bartenschlager R . Host-directed therapies for bacterial and viral infections. Nat Rev Drug Discov. (2018) 17:35–56. doi: 10.1038/nrd.2017.162, PMID: 28935918 PMC7097079

[28] Nisini R Poerio N Mariotti S De Santis F Fraziano M . The multirole of liposomes in therapy and prevention of infectious diseases. Front Immunol. (2018) 9. doi: 10.3389/fimmu.2018.00155, PMID: 29459867 PMC5807682

[29] Yeung T Grinstein S . Lipid signaling and the modulation of surface charge during phagocytosis. Immunol Rev. (2007) 219:17–36. doi: 10.1111/j.1600-065X.2007.00546.x, PMID: 17850479

[30] Honigmann A Van Den Bogaart G Iraheta E Risselada HJ Milovanovic D Mueller V . Phosphatidylinositol 4,5-bisphosphate clusters act as molecular beacons for vesicle recruitment. Nat Struct Mol Biol. (2013) 20:679. doi: 10.1038/nsmb.2570, PMID: 23665582 PMC3676452

[31] Pizarro-Cerdá J Charbit A Enninga J Lafont F Cossart P . Manipulation of host membranes by the bacterial pathogens Listeria, Francisella, Shigella and Yersinia. Semin Cell Dev Biol. (2016) 60:155–67. doi: 10.1016/j.semcdb.2016.07.019, PMID: 27448494 PMC7082150

[32] Terebiznik MR Vieira OV Marcus SL Slade A Yips CM Trimble WS . Elimination of host cell Ptdlns(4, 5)P2 by bacterial SigD promotes membrane fission during invasion by Salmonella. Nat Cell Biol. (2002) 4:766–73. doi: 10.1038/ncb854, PMID: 12360287

[33] Laencina L Dubois V Le Moigne V Viljoen A Majlessi L Pritchard J . Identification of genes required for Mycobacterium abscessus growth *in vivo* with a prominent role of the ESX-4 locus. Proc Natl Acad Sci United States America. (2018) 115:E1002–11. doi: 10.1073/pnas.1713195115, PMID: 29343644 PMC5798338

[34] Nguyen TQ Heo BE Jeon S Ash A Lee H Moon C . Exploring antibiotic resistance mechanisms in Mycobacterium abscessus for enhanced therapeutic approaches. Front Microbiol. (2024) 15. doi: 10.3389/fmicb.2024.1331508, PMID: 38380095 PMC10877060

[35] Johansen MD Herrmann JL Kremer L . Non-tuberculous mycobacteria and the rise of Mycobacterium abscessus. Nat Rev Microbiol. (2020) 18:392–407. doi: 10.1038/s41579-020-0331-1, PMID: 32086501

[36] Sánchez-Borges M Thong B Blanca M Ensina LFC González-Díaz S Greenberger PA . Hypersensitivity reactions to non beta-lactam antimicrobial agents, a statement of the WAO special committee on drug allergy. World Allergy Organ J. (2013) 6:18. doi: 10.1186/1939-4551-6-18, PMID: 24175948 PMC4446643

[37] Di Pietro C Zhang PX O’Rourke TK Murray TS Wang L Britto CJ . Ezrin links CFTR to TLR4 signaling to orchestrate anti-bacterial immune response in macrophages. Sci Rep. (2017) 7:10882. doi: 10.1038/s41598-017-11012-7, PMID: 28883468 PMC5589856

[38] Oviedo-Boyso J Cortés-Vieyra R Huante-Mendoza A Yu HB Valdez-Alarcón JJ Bravo-Patiño A . The phosphoinositide-3-kinase–akt signaling pathway is important for staphylococcus aureus internalization by endothelial cells. Infect Immun. (2011) 79:4569. doi: 10.1128/IAI.05303-11, PMID: 21844240 PMC3257907

[39] Zolin A Adamoli A Bakkeheim E van Rens J . ECFSPR Annual Report 2022. (2024). https://www.ecfs.eu/sites/default/files/Annual%20Report_2022_vs1.0_ECFSPR_20250130.pdf (Accessed October 29, 2024).

