# Supplementary material for: Phosphatidylserine liposomes for Mycobacterium abscessus infections management in people with cystic fibrosis non-eligible for CFTR modulators
Source: Front Immunol. 2026 Jan 28;17:1681558. doi: 10.3389/fimmu.2026.1681558 (PMC12891076; doi:10.3389/fimmu.2026.1681558)
Supplement: Supplementary file 1 [file Table1.docx]

Supplementary Material

Phosphatidylserine liposomes for *Mycobacterium abscessus* infections management in people with cystic fibrosis non-eligible for CFTR modulators

Tommaso Olimpieri^1^, Noemi Poerio^1^, Fabio Saliu^2^, Nicola I. Lorè^2^, Fabiana Ciciriello^3^, Greta Ponsecchi^1,^, Federico Alghisi^3^, Daniela M. Cirillo^2^, Marco M. D’Andrea^1^, and Maurizio Fraziano^1,*^

^1^Department of Biology, University of Rome Tor Vergata, Italy;

^2^Emerging Bacteria Pathogens Unit, San Raffaele Scientific Institute, Milan, Italy;

^3^Pneumology and Cystic Fibrosis Unit, Bambino Gesù Children’s Hospital, IRCCS, Rome, Italy;

*** Correspondence:**Maurizio Fraziano, Department of Biology, Tor Vergata University of Rome, Via della Ricerca Scientifica 1, 00133, Rome, Italy.
fraziano@bio.uniroma2.it

Keywords: Liposomes, Host-directed therapy, Cystic Fibrosis, *Mycobacterium abscessus*, Innate Immunity.

# Supplementary Data

Materials and Methods.
All experimental conditions can be found in “Materials and Methods” section of the main article.

# Supplementary Figures and Tables

## Supplementary Figures

Supplementary Figure 1.
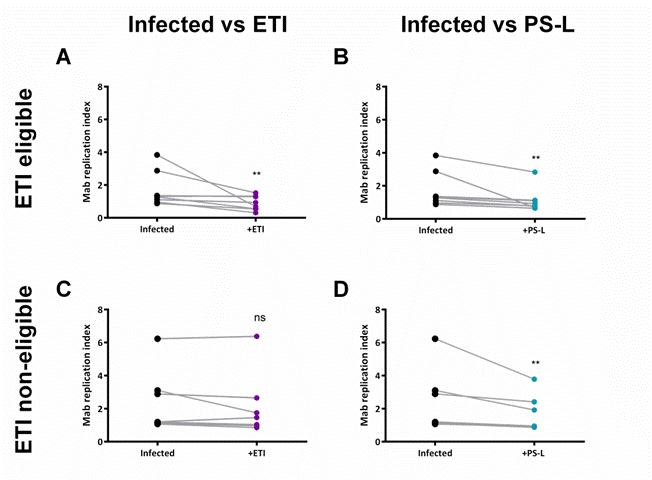
**PS-L treatment can reduce Mab intracellular viability in macrophages from pwCF irrespectively of their eligibility for the drug**

MDM form pwCF either eligible (n=8) (A and B) or non-eligible (n=7) (C and D) to ETI regimen were cultured at the concentration of 1x10^6^ cells/ml in 96-well plates. Cells were infected or not with Mab at MOI 10 for 3 hours at 37°C and then extracellular bacilli were killed by 1 hour incubation with amikacin 250µg/ml. Cells were finally stimulated or not with PS-L (B and D) or ETI (A and C). Replication index was calculated as the ratio between the CFU obtained after 18 hours from infection, in the presence or absence of stimuli, and those obtained immediately after the infection. Statistical analysis was performed by using two-sided Wilcoxon matched-pairs signed rank test. ns = not significant; ** p<0.01.

## Supplementary Tables


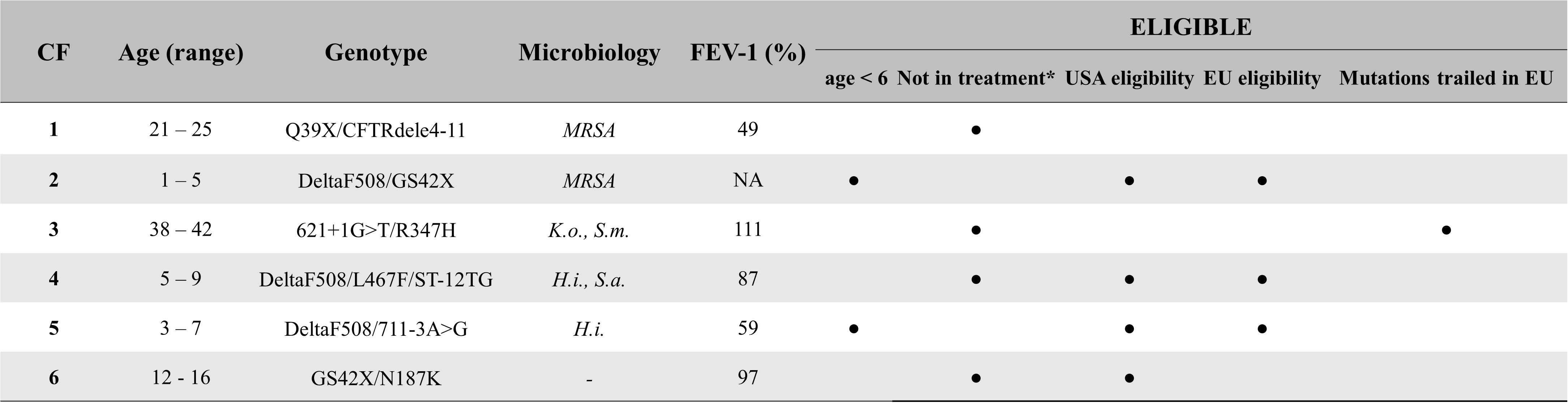
Supplementary Table 1. **Demographic and clinical characteristics of pwCF in Figure 1**

*Abbreviations:* ***H.i.****: Haemophilus influenzae;* ***K.o.****: Klebsiella oxytoca;* ***MRSA****: Methicillin-resistant Staphylococcus aureus;* ***S.m.****: Serratia marcescens;* ***S.a.****: Staphylococcus aureus;* **N/A**: not applicable: pwCF unable to safely perform spirometry because of their age; **Not in treatment***: eligible pwCF for ETI regimen either waiting for the prescription or refusing the medication.

Supplementary Table 2A. **Demographic and clinical characteristics of pwCF in Figure 2A**


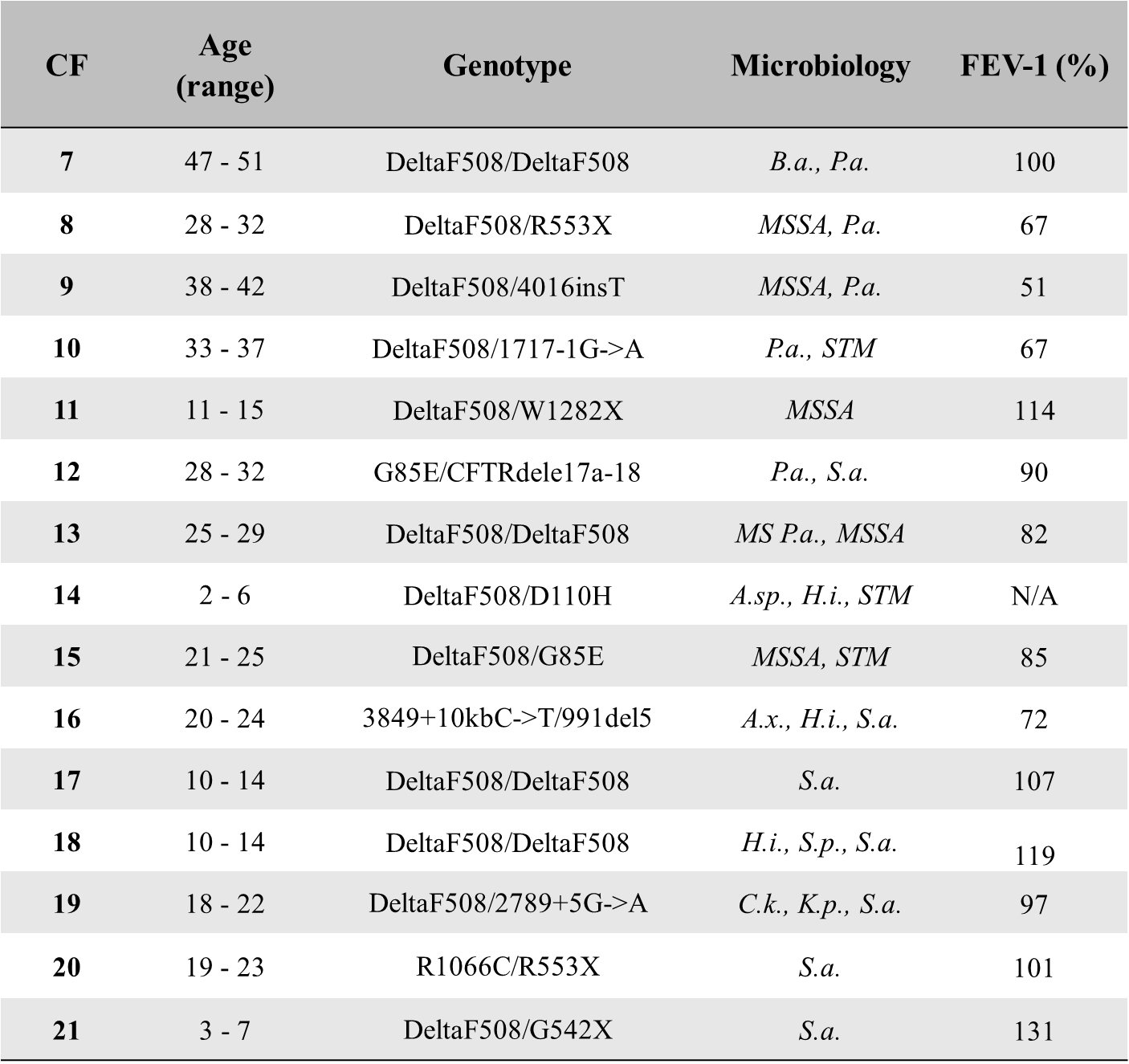


*Abbreviations:* ***A.x.****: Achromobacter xylosoxidans;* ***A.sp.****: Acinetobacter species;* ***B.a.****: Bramella caratarralis;* ***C.k.****: Citrobacter koseri;* ***H.i.****: Haemophilus influenzae;* ***K.p.****: Klebsiella pneumoniae;* ***MS P.a.****: Pseudomonas aeruginosa Mucoid Strain;* ***MSSA****: Methicillin-sensitive Staphylococcus aureus;* ***P.a.****: Pseudomonas aeruginosa;* ***S.a.****: Staphylococcus aureus;* ***S.p.****: Streptococcus pneumoniae;* ***STM****: Stenotrophomonas maltophilia;* **N/A**: not applicable: pwCF unable to safely perform spirometry because of their age;

**
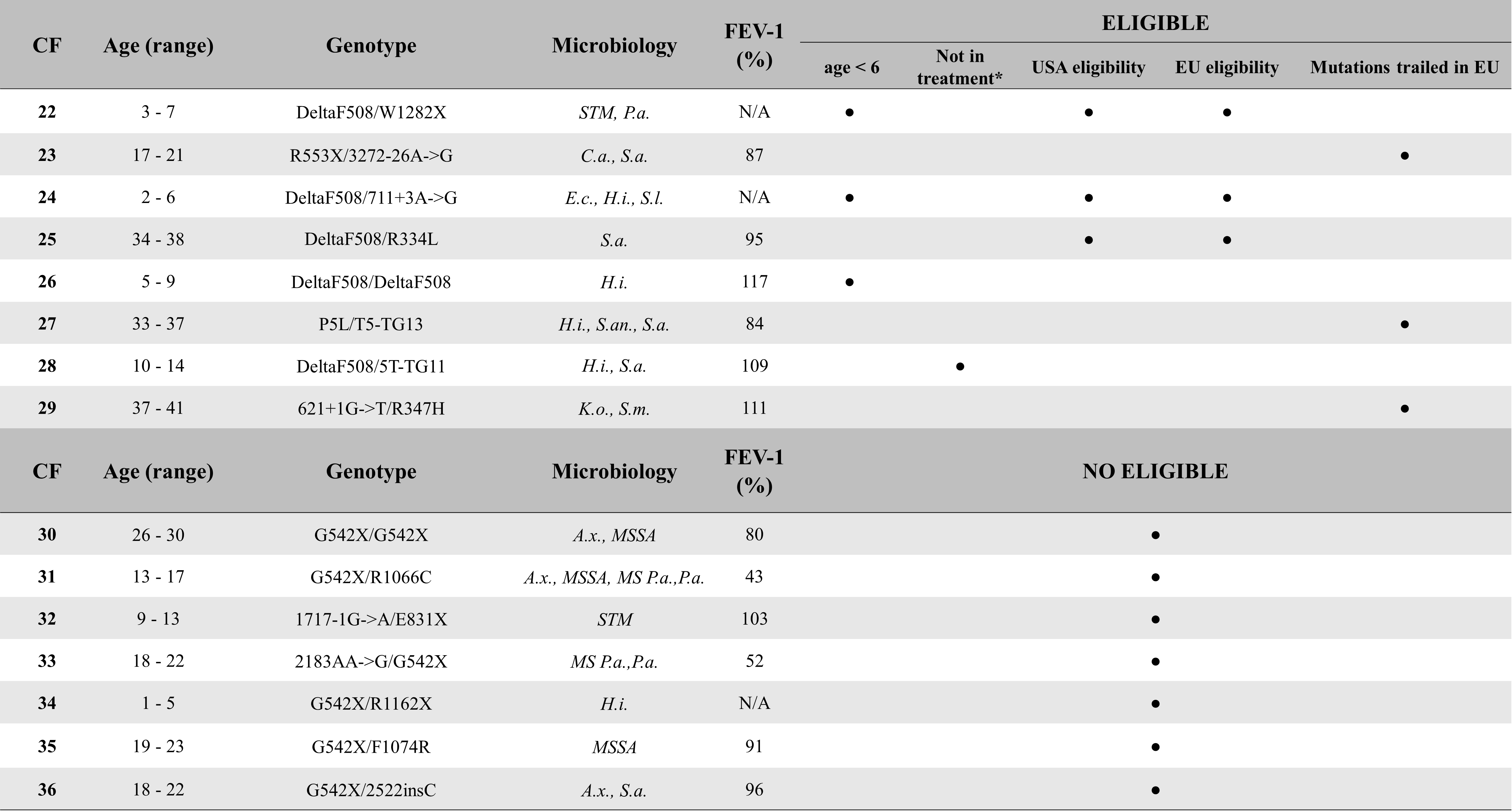
**Supplementary Table 2B. **Demographic and clinical characteristics of pwCF in Fig. 2B and Fig. S1**

*Abbreviations:* ***A.x.****: Achromobacter xylosoxidans;* ***C.a.****: Candida albicans;* ***E.c.****: Escherichia coli;* ***H.i.****: Haemophilus influenzae;* ***K.o.****: Klebsiella oxytoca;* ***MS P.a.****: Pseudomonas aeruginosa Mucoid Strain;* ***MSSA****: Methicillin-sensitive Staphylococcus aureus;* ***P.a.****: Pseudomonas aeruginosa;* ***S.l.****: Serratia liquefaciens;* ***S.m.****: Serratia marcescens;* ***S.a.****: Staphylococcus aureus;* ***S.an.****: Streptococcus anginosus;* ***STM****: Stenotrophomonas maltophilia;* **N/A**: not applicable: pwCF unable to safely perform spirometry because of their age; **Not in treatment***: eligible pwCF for ETI regimen either waiting for the prescription or refusing the medication.


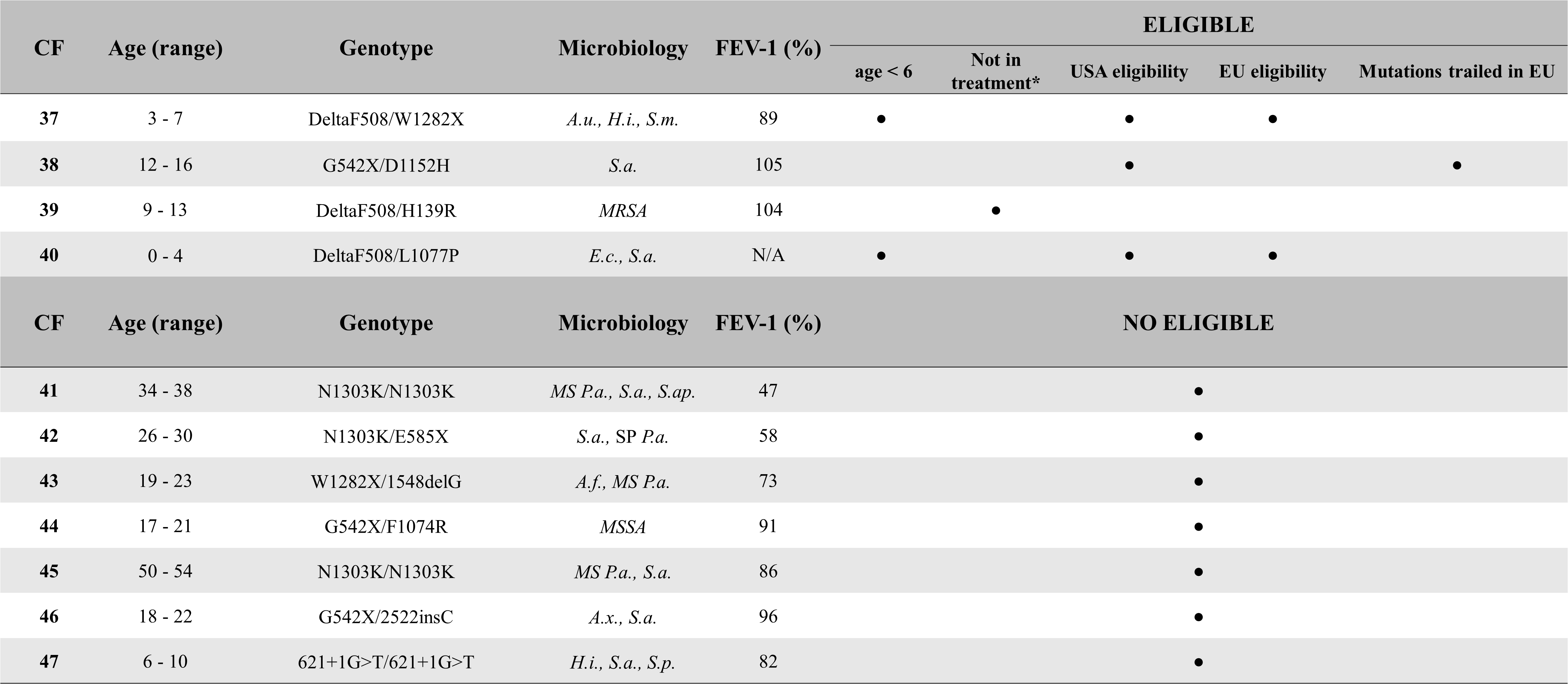
Supplementary Table 3. **Demographic and clinical characteristics of pwCF in Figure 3**

*Abbreviations:* ***A.f.****: Aspergillus fumigatus;* ***A.u.****: Acinetobacter ursingii;* ***A.x.****: Achromobacter xylosoxidans;* ***E.c.****: Escherichia coli;* ***H.i.****: Haemophilus influenzae;* ***MS P.a.****: Pseudomonas aeruginosa Mucoid Strain;* ***MSSA****: Methicillin-sensitive Staphylococcus aureus;* ***MRSA****: Methicillin-resistant Staphylococcus aureus;* ***S.ap.****: Scedosporium apiospermum;* ***S.m.****: Serratia marcescens;* ***S.a.****: Staphylococcus aureus;* ***S.p.****: Streptococcus pneumoniae;* ***SP P.a.****: Pseudomonas aeruginosa Small Phenotype;* **N/A**: not applicable: pwCF unable to safely perform spirometry because of their age; **Not in treatment***: eligible pwCF for ETI regimen either waiting for the prescription or refusing the medication.
